# Suicidal Thoughts and Behaviors Among Autistic Transgender or Gender-Nonconforming US College Students

**DOI:** 10.1001/jamanetworkopen.2024.38345

**Published:** 2024-10-09

**Authors:** Annabelle M. Mournet, John K. Kellerman, Roscoe C. Garner, Evan M. Kleiman

**Affiliations:** 1 Department of Psychology, Rutgers, The State University of New Jersey, Piscataway

## Abstract

**Importance:**

Suicide risk is a global public health crisis, with suicide ranking as a consistent leading cause of death among adults in the US. Autistic individuals and transgender or gender-nonconforming (TGNC) individuals represent populations with notably elevated rates of suicidal thoughts and behaviors (STBs).

**Objective:**

To characterize suicidal thoughts and behaviors among TGNC and autistic individuals, using a large, nationally representative sample.

**Design, Setting, and Participants:**

This study is a secondary analysis of cross-sectional data from students at colleges and universities throughout the US who participated in the American College Health Association National College Health Assessment from 2019 to 2023.

**Exposures:**

Autistic and TGNC identities were self-reported by participants.

**Main Outcomes and Measures:**

The frequency of intersectionality of autism and TGNC identities and whether those who had intersectional marginalized identities had increased likelihood of STBs were examined. STBs were self-reported by participants. A series of moderated regression analyses were performed to examine how the interaction between autism and possessing a marginalized gender identity (ie, TGNC status) was associated with STBs.

**Results:**

The sample included 41 507 college students with a mean (SD) age of 23.35 (6.83) years. A total of 2410 participants (5.81%) identified as being TGNC. Overall, 326 TGNC participants (13.53%) also identified as autistic, whereas 625 of those who identified as cisgender (1.58%) also identified as autistic. Gender identity and autism were associated with greater odds of STBs. For suicidal ideation, gender identity had an odds ratio (OR) of 3.34 (95% CI, 2.99-3.73), and autism had an OR of 2.06 (95% CI, 1.76-2.42). For suicide attempts, gender identity had an OR of 2.74 (95% CI, 2.13-3.52), and autism had an OR of 2.39 (95% CI, 1.62-3.52). A significant interaction existed for attempts (OR, 0.51; 95% CI, 0.27-0.97); nonautistic cisgender individuals had the lowest attempt rate.

**Conclusions and Relevance:**

This cross-sectional study addresses the dearth of information on how intersectionality in gender and autism status impacts the risk of STBs, and the results confirm the elevated risk of STBs among TGNC and autistic populations. Interventions are needed to support college students with these identities.

## Key Points

**Question:**

How is the interaction of autism and transgender or gender-nonconforming (TGNC) identities associated with the risk of suicidal thoughts and behaviors (STBs) among college students?

**Findings:**

In this cross-sectional study of 41 507 US college students, there was significant overlap in autism and TGNC identities. Moreover, gender identity and autism were both associated with greater odds of STBs, and a significant interaction existed for suicide attempts.

**Meaning:**

These findings underscore the need for interventions to support TGNC and autistic college students, who are at greater risk for STBs, including through campuswide initiatives.

## Teaser

This cross-sectional study characterizes the associations of transgender or gender-nonconforming identity and autism with suicidal thoughts and behaviors among a large, nationally representative sample of US college students.

## Introduction

Suicide risk is a global public health crisis, with suicide ranking as a consistent leading cause of death among adults in the US.^1^ Autistic individuals and transgender or gender-nonconforming (TGNC) individuals represent populations with notably elevated rates of suicidal thoughts and behaviors (STBs). A recent national probability sample found strikingly high lifetime rates of STBs among TGNC adults: 81.3% of TGNC participants reported suicidal ideation in their lifetime, and 42.0% reported they had previously attempted suicide.^2^ Elevated rates of interpersonal and structural discrimination, limited access to health care, and mental health comorbidities are factors that may contribute to the disparately high STB rates among TGNC individuals.^3,4^ In addition, previous research^5^ has highlighted increased rates of involvement in risk behaviors (ie, substance use, sexual behavior, emotional distress, and bullying victimization) along with lower levels of protection among TGNC young people compared with their cisgender counterparts. In addition to risk among TGNC individuals, research has demonstrated that autistic individuals represent a population with a high risk of STBs.^6^ In one study^7^ of suicide risk among autistic adults, 66% of participants reported suicidal ideation and 35% reported a lifetime history of suicide plans or suicide attempts, going beyond what was largely anecdotal evidence of a disparity in the rate of suicide risk among autistic individuals compared with nonautistic individuals.

Beyond the elevated risk associated with each of these distinct communities, it is essential to consider the demonstrated overlap in identification with these 2 intersectional identities. Indeed, more than 1 in 4 TGNC people also meet the criteria for autism.^8^ Studies of gender variance among young people have similarly detected that autistic individuals were 7.76 times more likely to report gender variance compared with nonautistic individuals.^9^ Despite research demonstrating that these 2 groups overlap considerably and that membership in either group is associated with increased risk for STBs, there has been very little work examining the impact of identifying with both of these populations on STBs. To date, 2 studies^10,11^ have begun to investigate suicide risk among autistic TGNC adults, primarily from the lens of understanding mental health concerns more broadly among this intersectional population. For instance, Strauss and colleagues^11^ found that among a sample of TGNC adolescents and young adults, autistic TGNC individuals were more likely to report STBs. A recent systematic review and meta-analysis^12^ sought to advance the understanding of rates of risk of suicide attempts among gender-diverse and neurodiverse populations, with findings that STB rates were greater among gender-diverse individuals compared with neurodiverse individuals; however, it should be noted that there was insufficient research to test suicide risk among individuals who are both gender-diverse and neurodiverse, underscoring a need for more research on this intersectional population.

College students, in particular, represent a population worth investigating in relation to these topics. Transgender college students have been shown to have approximately twice the risk for most mental health conditions compared with cisgender students.^13^ TGNC college students also report greater rates of STBs compared with cisgender students, with suicide attempt rates approximately 3 times higher among transgender students.^14^ Identity-specific stressors, such as lack of access to gender-affirming housing and bathroom options, are associated with increased risk for STBs among TGNC college students.^15^ Moreover, the importance of supporting the mental health of autistic college students has also been supported,^16^ with recent efforts to provide clearer resources to this population.^17^ Finally, college students are also a population with high rates of STBs.^18^ Taken together, this existing research suggests that college students represent an ideal population to study suicide risk among TGNC autistic individuals. Therefore, we aim to characterize and describe suicidal thoughts and behaviors among TGNC autistic individuals, using a large, nationally representative sample of college students.

## Methods

### Study Design and Participants

This cross-sectional study uses wave III data from the American College Health Association-National College Health Assessment, which surveyed undergraduate and graduate students from fall 2019 to spring 2023.^19,20^ Colleges and universities throughout the US administer the survey in either the fall or spring semester using multiple administration methods (eg, online and paper) and sampling strategies. Participant demographic characteristics, including class year, are reported by wave in Table 1. This study was exempt from institutional review board approval because of the use of secondary data exclusively. Participants provided consent by agreeing to complete the survey. This report follows the Strengthening the Reporting of Observational Studies in Epidemiology (STROBE) reporting guideline for observational studies.

**Table 1.** Demographic Characteristics Across Wave

Characteristic	Participants, No. (%) (N = 41 507)
Age, mean (SD), y	23.35 (6.83)
Class year	
First year	7748 (18.68)
Second year	6845 (16.50)
Third year	8664 (22.89)
Fourth year	6974 (16.81)
Fifth year	2240 (5.40)
Master’s student	4575 (11.03)
Doctoral student	3648 (8.79)
Other	792 (1.91)
Missing	21 (0.05)
Race and ethnicity^a^	
American Indian or Alaska Native	903 (2.18)
Asian	6026 (14.52)
Black	2398 (5.78)
Hispanic or Latinx	7236 (17.43)
Middle Eastern	789 (1.90)
Native Hawaiian	252 (0.61)
White	26 773 (64.50)
Biracial or multiracial	1909 (4.60)
Any other identity not otherwise specified	658 (1.59)

^a^ Race categories add up to greater than 100% because participants had the option to select multiple categories.

### Measures

#### Demographics

Participants responded to items about their gender identity, sexual orientation, age, class year, and race and ethnicity. For gender, participants were asked, “Which term do you use to describe your gender identity?” Response options are listed in Table 2. For race and ethnicity, participants were able to select multiple options. Individuals who selected multiple races were recoded into the category of biracial or multiracial. Table 1, which provides demographic information on age, class year, race, and ethnicity, lists the individual response options. Data on race and ethnicity were included in this study to characterize the demographics of the sample and because of the importance of intersectionality with regard to suicide risk.

**Table 2.** Autism Diagnosis by Gender Identity

Gender identity	Participants, No. (%)		Total No. (N = 41 954)
	Autistic (n = 951 [2.27%])	Nonautistic (n = 41 003 [97.73%])	
Cisgender total	625 (1.58)	38 919 (98.42)	39 544
Cisgender man^a^	298 (2.46)	11 834 (97.54)	12 132
Cisgender woman^b^	327 (1.19)	27 085 (98.81)	27 412
Transgender and gender-nonconforming total	326 (13.53)	2084 (86.47)	2410
Transgender man^c^	24 (20.34)	94 (79.64)	118
Transgender woman^c^	14 (27.45)	37 (72.55)	51
Nonbinary^c^	147 (18.10)	665 (81.90)	812
Genderfluid^c^^,^^d^	37 (17.70)	172 (82.30)	209
Genderqueer^c^^,^^d^	35 (17.86)	161 (82.14)	196
Agender^c^	32 (29.63)	76 (70.37)	108
Other^c^^,^^d^	37 (18.88)	159 (81.12)	196

^a^ Cisgender men were considered the reference.

^b^ *P* < .05 vs cisgender men.

^c^ All transgender and gender-nonconforming categories were not significantly different from each other but were significantly different from cisgender men (*P* < .05).

^d^ Genderfluid, genderqueer, and other identities were not significantly different from each other but were significantly different from cisgender men (*P* < .05).

#### Autism Status

Participants were asked, “Do you have any of the following?” with “autism spectrum disorder” provided as 1 of 7 listed conditions. Response options were yes and no.

#### Past-Year STBs

Participants were asked, “Within the last 12 months, have you attempted suicide?” with response options of yes and no. Participants were asked, “How often have you thought about killing yourself in the past year?” with response options of never, rarely (1 time), sometimes (2 times), often (3-4 times), and very often (≥5 times). To be consistent with the binary format of past-year suicide attempts, never was recoded as no past-year suicidal ideation, and all other endorsements were collapsed to past-year suicidal ideation.

### Statistical Analysis

An a priori significance level of *P* < .05 was set. All hypothesis testing was 2-sided.

#### Prevalence of Intersectional Marginalized Identities

We calculated the percentage of individuals who have an autism diagnosis across each gender identity listed in Table 2. To examine significant differences in proportions of autism across different gender categories, we conducted a high-level χ^2^ analysis for the cisgender vs TGNC interaction with autism vs no autism. For a more granular perspective, we conducted an omnibus χ^2^ analysis to examine proportions of each gender identity having an autism diagnosis. The omnibus test was followed by post hoc pairwise comparisons (ie, 1 *df* χ^2^ tests) to test for differences in autism diagnosis between each gender identity.

#### Associations Between Intersectional Marginalized Identities and STBs

We conducted a series of moderated regression analyses examining how the interaction between having autism and possessing a marginalized gender identity (ie, TGNC status) was associated with suicidal ideation and suicide attempts. We conducted 1 model per outcome (ideation and attempts). A multiple regression using dummy coding, with the no marginalized identity group as the reference, was used to probe significant interactions. Because data were nested within schools, all logistic regression models were multilevel logistic regression models, conducted in the lme4 package in R statistical software version 4.3.2 (R Project for Statistical Computing) with fixed slopes (because we had no a priori hypotheses that would necessitate random slopes). Moreover, given the potential heterogeneity in risk for STBs across each of the 9 gender identities that could be endorsed, we also conducted supplemental analyses examining the association between each individual gender and suicidal thoughts and suicide attempts. These analyses were exploratory in nature as a result of insufficient prior research on which to base hypotheses. Supplemental analyses were performed controlling for race and are shown in eTable in Supplement 1.

## Results

The sample included 41 507 college students with a mean (SD) age of 23.35 (6.83) years; 39 544 participants (95.27%) identified as cisgender and 2410 (5.81%) participants identified as gender minorities. Table 1 summarizes additional demographic information of the sample.

### Describing Prevalence of Intersectional Marginalized Identities

Overall, 326 (13.53%) of those who identified as TGNC also identified as autistic, whereas only 625 (1.58%) of those who identified as cisgender also identified as autistic. The omnibus χ^2^ examining individual gender identities revealed significant differences in autism diagnosis by gender. The proportion of individuals with an autism diagnosis ranged from 1.19% (327 individuals) among cisgender women to 29.63% (32 individuals) among agender individuals. Pairwise comparisons are reported in Table 2 and depict significant differences in autism diagnoses by gender identity.

### Association Between Intersectional Marginalized Identities and Suicidal Ideation

#### Primary Analyses Examining Gender Identity and Autism Interaction

There were significant positive main effects on suicidal ideation for both gender identity (TGNC vs not) (odds ratio [OR], 3.34; 95% CI, 2.99-3.73; *P* < .001) and autism (OR, 2.06; 95% CI, 1.76-2.42; *P* < .001). Regarding interaction effects, there was no significant interaction between gender identity and autism on suicidal ideation (OR, 0.78; 95% CI, 0.57-1.06; *P* = .11) (Table 3).

**Table 3.** Interaction Effects for Gender Identity and Autism Status Association With Suicidal Thoughts and Behaviors

Variable	Suicidal ideation^a^		Suicide attempts^b^	
	OR (95% CI)	*P* value	OR (95% CI)	*P* value
Intercept	0.46 (0.43-0.50)	<.001	0.02 (0.02-0.02)	<.001
Gender identity (transgender and gender-nonconforming vs cisgender)	3.34 (2.99-3.73)	<.001	2.74 (2.13-3.52)	<.001
Autistic vs nonautistic	2.06 (1.76-2.42)	<.001	2.39 (1.62-3.52)	<.001
Interaction of gender identity with autism	0.78 (0.57-1.06)	.11	0.51 (0.27-0.97)	.04

Abbreviation: OR, odds ratio.

^a^ Suicidal ideation model marginal *R*^2^ = 0.022.

^b^ Suicide attempt model marginal *R*^2^ = 0.016.

#### Supplemental Analyses Considering Relative Risk for Suicidal Ideation by Individual Gender

When examining supplemental analyses looking at individual genders, compared with cisgender men, all other gender identities had statistically significantly greater odds of suicidal ideation. The ORs ranged from 1.08 (95% CI, 1.03-1.13) for cisgender women to 4.29 (95% CI, 2.90-6.34) for agender individuals (Table 4).

**Table 4.** Exploratory Analyses for Effects for Gender Identity Association With Suicidal Thoughts and Behaviors

Gender^a^	Suicidal ideation		Suicide attempts	
	OR (95% CI)	*P* value	OR (95% CI)	*P* value
Cisgender woman	1.08 (1.03-1.13)	.002	0.71 (0.62-0.83)	<.001
Transgender man	4.09 (2.83-5.90)	<.001	2.44 (1.11-5.34)	.03
Transgender woman	3.62 (2.14-6.15)	<.001	4.39 (1.73-11.14)	.002
Nonbinary	4.25 (3.66-4.94)	<.001	2.14 (1.53-2.98)	<.001
Genderfluid	4.02 (3.03-5.35)	<.001	2.17 (1.16-4.05)	.02
Genderqueer	3.61 (2.71-4.81)	<.001	1.86 (0.95-3.66)	.07
Agender	4.29 (2.90-6.34)	<.001	3.60 (1.79-7.25)	<.001
Other	2.58 (1.95-3.42)	<.001	2.11 (1.11-4.00)	.02

Abbreviation: OR, odds ratio.

^a^ Reference group for gender is cisgender man.

### Association Between Intersectional Marginalized Identities and Suicide Attempts

#### Primary Analyses Examining Gender Identity and Autism Interaction

There were significant positive main effects on suicide attempts for both gender identity (TGNC vs not) and autism. There was a significant interaction between gender identity and autism for suicide attempts (Table 3). A follow-up probe of the interaction using dummy coding and the reference group set as nonautistic cisgender revealed that autistic cisgender individuals (OR, 2.39; 95% CI, 1.62-3.52; *P* < .001), nonautistic TGNC individuals (OR, 2.74; 95% CI, 2.13-3.52; *P* < .001), and autistic TGNC individuals (OR, 3.35; 95% CI, 2.11-5.30; *P* < .001) all had significantly elevated risk of suicide attempts compared with the nonautistic cisgender group. Autistic TGNC participants had the highest risk of suicide attempts, although there was no significant difference between this group and the autistic cisgender individuals and nonautistic TGNC individuals, given overlapping 95% CIs and point estimates that fit within the 95% CIs of other effects (Table 3).

#### Exploratory Analyses Considering Relative Risk for Suicide Attempts by Individual Gender

Cisgender women had decreased odds of suicide attempts compared with cisgender men, although most other gender identities had greater odds of suicide attempts compared with cisgender men. Genderqueer individuals did not have significantly different odds of suicide attempts compared with cisgender men (Table 4).

## Discussion

Using a large sample of college students across the US, this cross-sectional study provides important information on the intersection between gender identity and autism diagnosis. The main effects from this study are consistent with existing research showing that TGNC and autistic individuals are both populations with greater odds of STBs. This study provides some of the very first evidence examining the impact of the intersection of both marginalized identity on suicidal ideation and suicide attempts, highlighting an interaction effect for suicide attempts among this understudied population. With increasing recognition of suicide risk among TGNC and autistic individuals, this work seeks to further this important area of research by addressing the dearth of research on how intersectionality in gender and neurodevelopmental status impacts risk.

Regarding the intersectionality of gender and autism, we found that cisgender women had the lowest rates of autism, followed by cisgender men. This may be reflective of the longstanding concern of underdiagnosis of autism among women.^21,22^ Transgender men and women, as well as nonbinary, genderqueer, genderfluid, and other gender identities, had rates of autism comparable to one another, although greater rates than cisgender men and women. Agender individuals had significantly greater rates of autism than all other genders. Although existing research has highlighted considerable overlap in autism and identifying as a gender minority,^23^ limited research has provided this level of granularity regarding autism across specific gender minority subgroups, such as agender individuals. Elevated rates of autism among agender individuals is a novel finding that requires further investigation to understand the overlap in these identities and underscores the importance of greater research in general that seeks to understand the mental health of agender individuals. Importantly, this study leverages a sample of college students. Although a college sample is advantageous given elevated risk for the outcomes of focus for this study, it is important that future research seek to replicate these findings across other samples.

Current findings support existing research regarding TGNC individuals^1^ and autistic individuals^6^ having greater odds of STBs compared with cisgender and nonautistic individuals. Research on the mental health of TGNC individuals is often critiqued for its failure to consider differences by subgroup due to limited sample sizes.^24^ For instance, an existing systematic review^25^ looking at TGNC individuals and mental health further acknowledged a lack of nationally representative sample of TGNC individuals as a limitation of existing research. Regarding the main effects of TGNC college students’ risk of STBs, given our large sample, we examined the risk of suicidal ideation and attempts by gender identity subgroup. Accordingly, we report ORs that are specific to each gender identity. Consistent themes emerged, with all gender minority identities having greater odds of both suicidal ideation and attempts compared with the reference group of cisgender men. Importantly, one exception to this is that genderqueer individuals did not have significantly different rates of suicide attempts compared with cisgender men. There is a lack of existing research on genderqueer individuals, preventing clear interpretation of this finding. Greater research is needed to better understand this finding and see whether it is replicable. Of note, we found that cisgender women were significantly more likely to report suicidal ideation compared with cisgender men but were significantly less likely to report suicide attempts compared with cisgender men. This finding is largely consistent with the gender paradox of suicide,^26^ in which women typically report higher levels of ideation, supporting the validity of our findings. Notably, our finding that cisgender women had decreased odds of suicide attempts compared with cisgender men is somewhat inconsistent with the gender paradox of suicide, which states that women typically have higher rates of suicidal ideation and behavior than men, whereas suicide deaths are typically lower for women than for men.^26^ Our results depict that women had lower endorsement of suicidal behaviors compared with cisgender men. This may point to differences in risk by age, given our focus on college students, which is consistent with existing literature on adolescents and young adults, where suicide death rates were higher in young men, while young women had higher rates of suicide attempts.^27^

Interaction effects revealed no significant interaction between gender and autism status for the outcome of suicidal ideation. For suicide attempts, the group with the lowest odds of suicidal behavior was nonautistic cisgender individuals. Autistic individuals and TGNC individuals were found to have elevated rates of suicide attempts; however, identifying as both having autism and being TGNC did not lead to significantly increased odds of suicide attempts compared with identifying with just one of these identities. However, there may be specific risk factors that contribute to STBs for TGNC autistic individuals, making additional research with this vulnerable group essential. Intervening on suicide risk for TGNC autistic individuals may present specific challenges and opportunities for treatment development and adaptations. Findings of a significant interaction for attempts but not ideation is in line with the recent push for research to better understand the distinction between ideation and attempts.^28^

### Limitations

The current findings should be considered in the context of the following limitations. Although this study uses a large sample of college students recruited over 4 years, owing to the sampling methods used, there may be repeated participants who were not linked with a consistent identifier. This prevented us from removing potential duplicate participants, which may impact the findings. Outcomes of interest were assessed using single-item questions, limiting the granularity of analyses. Further exploring these questions with a more nuanced report of STBs may be helpful to elucidate relationships among autism, gender identity, and different dimensions of suicide risk. In addition, autism was also assessed with a single, self-reported item. Future research may benefit from investigation into optimal methods to assess autism diagnosis in self-report questionnaires studies. In addition, this study features a sample that is predominantly White in terms of race. Importantly, autism status in this study is queried in a manner such that both people who have received a formal diagnosis and those who have not received a formal diagnosis can respond affirmatively. Given disparities in access to diagnosis across gender and race,^29^ this may lead to increased diversity in the autistic sample of this study compared with other studies. Nonetheless, future research should look to expand examination of suicidal thinking and behaviors by leveraging racially diverse communities of TGNC autistic individuals.

## Conclusions

This study is innovative in examining the intersection between gender and autism and the impact of these shared identities on suicide risk, doing so in a large sample. Accordingly, this study provides essential information for supporting the mental health of college students. The main effects are consistent with existing literature and descriptive findings, and the interaction effects provide novel information on these overlapping identities and their risk of STBs. These findings underscore the need for interventions to support autistic TGNC college students, who may be more likely to experience and report STBs. For instance, campuswide efforts are needed to address risk. Moreover, the results from this study have the potential to go beyond college student risk, advocating for the importance of policies that seek to reduce risk of STBs among these high-risk populations. Numerous avenues for future research arise as well, particularly the need to advance the understanding of the mental health of agender and genderqueer individuals.

## Notes

Supplement 1. **eTable.** Interaction effects for gender identity and autism status relationship with STBs controlling for race/ethnicity

Supplement 2.
Data Sharing Statement
