# Supplementary material for: Suicidal Thoughts and Behaviors Among Autistic Transgender or Gender-Nonconforming US College Students
Source: JAMA Netw Open. 2024 Oct 9;7(10):e2438345. doi: 10.1001/jamanetworkopen.2024.38345 (PMC11465100; doi:10.1001/jamanetworkopen.2024.38345)
Supplement: Supplement 1. — eTable. Interaction effects for gender identity and autism status relationship with STBs controlling for race/ethnicity [file jamanetwopen-e2438345-s001.pdf]

Supplemental Online Content

Mournet AM, Kellerman JK, Garner RC, Kleiman EM. Suicidal thoughts and behaviors among autistic transgender or gender-nonconforming US college students. *JAMA Netw Open*. 2024;7(10):e2438345. doi:10.1001/jamanetworkopen.2024.38345

eTable. Interaction effects for gender identity and autism status relationship with STBs controlling for race/ethnicity

This supplemental material has been provided by the authors to give readers additional information about their work.

© 2024 Mournet AM et al. *JAMA Network Open.*

eTable: Interaction effects for gender identity and autism status relationship with STBs controlling for race/ethnicity

	Suicidal ideation		Suicide attempts	
**Variable**	*Odds ratio (95% CI)*	*p-value*	*Odds ratios (95% CI)*	*p-value*
(Intercept)	0.4 (0.30-0.52)	<.001	0.04 (0.02-0.08)	<.001
Gender identity (TGNC vs. cisgender)	3.3 (2.95-3.69)	<.001	2.77 (2.15-3.56)	<.001
Autism vs. non-autistic	2.04 (1.73-2.39)	<.001	2.45 (1.66-3.62)	<.001
Asian	1.12 (0.84-1.49)	.427	0.4 (0.21-0.76)	.005
Biracial	1.63 (1.22-2.18)	.001	0.66 (0.34-1.29)	.228
Black / African American	1.14 (0.85-1.53)	.388	0.66 (0.34-1.29)	.227
Caucasian	1.18 (0.90-1.56)	.234	0.42 (0.23-0.78)	.006
Native Hawaiian / Pacific Islander	1.68 (1.05-2.67)	.029	0.76 (0.23-2.54)	.661
Hispanic	1.02 (0.77-1.35)	.896	0.46 (0.24-0.88	.019
Middle Eastern / North African or Arab Origin	1.04 (0.74-1.46)	.827	0.46 (0.19-1.11)	.084
Other	1.09 (0.79-1.50)	.609	0.37 (0.16-0.87)	.022
Gender identity X autism	0.77 (0.57-1.05)	.099	0.47 (0.25-0.91)	.025
**Random Effects**				
σ^2	3.29		3.29	
τ_{00} PERMID	0.05		0.01	
ICC	0.02		0	
N PERMID	54		54	
Observations	41055		40908	
Marginal R^2 / Conditional R^2	0.025 / 0.040		0.023 / 0.026	

© 2024 Mournet AM et al. *JAMA Network Open.*
