# Supplementary material for: Suicidal Thoughts and Behaviors Among Autistic Transgender or Gender-Nonconforming US College Students
Source: JAMA Netw Open. 2024 Oct 9;7(10):e2438345. doi: 10.1001/jamanetworkopen.2024.38345 (PMC11465100; doi:10.1001/jamanetworkopen.2024.38345)
Supplement: Supplement 2. — Data Sharing Statement [file jamanetwopen-e2438345-s002.pdf]

Data Sharing Statement

Mournet. Suicidal Thoughts and Behaviors Among Autistic Transgender or Gender-Nonconforming US College Students. *JAMA Netw Open*. Published October 09, 2024. doi:10.1001/jamanetworkopen.2024.38345

Data
Data available: Yes
Data types: Other (please specify)
Additional Information: The study uses existing data available from ACHA's NCHA and is available upon request from ACHA.
How to access data: Acknowledgements highlight how to access this data.
When available: With publication

Supporting Documents
Document types: None

Additional Information
Who can access the data: Data is available upon request to ACHA.
Types of analyses: Data is available upon request to ACHA.
Mechanisms of data availability: Data is available upon request to ACHA.
